# DIMINISHED RETURNS OF PARENTAL EDUCATION ON COGNITION: EVIDENCE FROM THREE POPULATION STUDIES IN THE US

**DOI:** 10.1093/geroni/igad104.1548

**Published:** 2023-12-21

**Authors:** Addam Reynolds, Emily Greenfield

**Affiliations:** University of Southern California, Los Angeles, California, United States; Rutgers, The State University of New Jersey, New Brunswick, New Jersey, United States

## Abstract

Mounting evidence suggests that the protective effects of one’s own higher socioeconomic status (SES) on health is diminished among minoritized racial/ethnic groups in the United States (U.S.) with respect to outcomes such as mortality, hospitalization and mental health. Extending this area of research to childhood SES and later life outcomes, this study examines whether the protective effects of higher parental education on cognition is diminished among Black adults relative to their non-Hispanic White peers. Using data from the Health and Retirement Study, the Midlife in the U.S. Study, and the National Social Life, Health and Aging Project, I examine whether associations between parental education and two measures of cognition (episodic memory and global cognition) are moderated by race (non-Hispanic White or Black) using a random-effects individual participant data meta-analysis approach. I find evidence for a small, but robust, protective effect of higher parental education on both episodic memory and global cognition among White adults. Among Black adults, there was no association between parental education and neither episodic memory nor global cognition. This study provides evidence that the protective effect of higher parental education on cognition is not the same across racialized populations, consistent with the theory of Minority Diminished Returns. As scholars continue calls for life course-oriented efforts to reduce racialized cognitive disparities, it is important to consider early-life risk and protective factors in the context of structural racism.

